# The effectiveness of Chance UK’s mentoring programme in improving behavioural and emotional outcomes in primary school children with behavioural difficulties: study protocol for a randomised controlled trial

**DOI:** 10.1186/s40359-018-0220-9

**Published:** 2018-03-12

**Authors:** Laura Whybra, Georgina Warner, Gretchen Bjornstad, Tim Hobbs, Lucy Brook, Zoe Wrigley, Vashti Berry, Obioha C. Ukoumunne, Justin Matthews, Rod Taylor, Tim Eames, Angeliki Kallitsoglou, Sarah Blower, Nick Axford

**Affiliations:** 1 Dartington Service Design Lab, Higher Mills, Buckfast Abbey, Buckfastleigh, TQ11 0EE UK; 2 Autistica, St Saviour’s House, 39-41 Union Street, London, SE1 1SD UK; 3 Peninsula Cerebra Research Unit (PenCRU), University of Exeter Medical School, St. Luke’s Campus, Heavitree Road, Exeter, EX1 2LU UK; 4 Depression and Anxiety Service, Sherborne House, Kingsteignton Road, Newton Abbot, TQ12 2PF UK; 5 School of Social Sciences, Cardiff University, Postgraduate Office, 1-3 Museum Place, Cardiff, CF10 3BD UK; 6 NIHR CLAHRC South West Peninsula (PenCLAHRC), University of Exeter, St. Luke’s Campus, Heavitree Road, Exeter, EX1 2LU UK; 7 University of Exeter Medical School, University of Exeter, St. Luke’s Campus, Heavitree Road, Exeter, EX1 2LU UK; 8 Exeter Clinical Trials Support Network, Royal Devon & Exeter Foundation NHS Trust, Barrack Road, Exeter, EX2 5DW UK; 9 School of Education, University of Roehampton, Roehampton Lane, London, SW15 5PJ UK; 10 Department of Health Sciences, University of York, Area 2 ATB/152 Seebohm Rowntree Building, Heslington, York, YO10 5DD UK; 11 NIHR CLAHRC South West Peninsula (PenCLAHRC), Plymouth University Peninsula Schools of Medicine and Dentistry, ITTC, Plymouth Science Park, Plymouth, PL6 8BX UK

**Keywords:** Mentoring, Behavioural and emotional problems, Randomised controlled trial, Children, Early intervention

## Abstract

**Background:**

There is a need to build the evidence base of early interventions to promote children’s health and development in the UK. Chance UK is a voluntary sector organisation based in London that delivers a 12-month mentoring programme for primary school children identified by teachers and parents as having behavioural and emotional difficulties. The aim of the study is to determine the effectiveness of the programme in terms of children’s behaviour and emotional well-being; this is the primary outcome of the trial.

**Methods/Design:**

A randomised controlled trial will be conducted in which participants are randomly allocated on a dynamic basis to one of two possible arms: the intervention arm (*n* = 123) will be offered the mentoring programme, and the control arm (*n* = 123) will be offered services as usual. Outcome data will be collected at three points: pre-intervention (baseline), mid-way through the mentoring year (c.9 months after randomisation) and post- mentoring programme (c.16 months after randomisation).

**Discussion:**

This study will further enhance the evidence for early intervention mentoring programmes for child behaviour and emotional well-being in the UK.

**Trial registration:**

Current Controlled Trials ISRCTN47154925. Retrospectively registered 9 September 2014.

## Background

Longitudinal research indicates that serious anti-social behaviour in adolescence and adulthood can be predicted by early signs of behavioural and emotional difficulties in childhood [1]. Individual-level risk factors for anti-social behaviour often express themselves as impulsiveness, difficulties in relating well to peers, poor problem-solving skills and an inability to regulate conduct and emotions [2, 3]. Left untreated, childhood behavioural and emotional difficulties, which affect approximately 10% of children aged 5–15 in Britain [4], elevate children’s risk for poor outcomes across multiple domains, including academic achievement, health, social relationships and offending [5–11]. It is therefore important to address selected individual and family risk factors in order to prevent behavioural and emotional difficulties in childhood and avert later anti-social and criminal behaviour.

### Realising ambition

Programmes that have been developed and tested in the US dominate the evidence base on what works to divert children and young people away from pathways into anti-social behaviour and crime. The UK is home to many innovative programmes, particularly in the charity sector, but few of these programmes have undergone the level of robust evaluation necessary to determine their impact on children’s outcomes [12]. In the light of several recent examples of programmes imported from the US proving to be largely ineffective in the UK [13, 14], it is important to develop home-grown interventions and test their effectiveness.

The Big Lottery Fund’s Realising Ambition programme seeks to build the evidence base for what works to prevent youth offending in the UK by funding the replication of home-grown and imported interventions with either proven or preliminary evidence of impact on child outcomes [15]. It involves a £25m investment over 5 years (2013–2017) in a portfolio of 25 interventions that are designed to intervene early in order to divert children and young people aged 8–14 away from pathways into crime. Chance UK’s early intervention mentoring programme for children aged 5–11 years with behavioural difficulties has been delivered in London for over 20 years and is one of the interventions selected for inclusion in the Big Lottery Realising Ambition portfolio.

### Mentoring to improve child outcomes

Mentoring programmes typically involve a supportive relationship between a child and positive adult role model who enables the child to take part in positive activities and make a commitment to socially appropriate goals. It is theorised that this contributes to children’s social-emotional, cognitive and identity development and that this acts as the mechanism through which mentoring has the potential to improve developmental outcomes, including behaviour [16].

Meta-analytic reviews indicate that mentoring typically reduces conduct problems, aggression and substance use [17, 18]. There are also reported improvements in educational achievement, social competence and emotional well-being [19–21]. Meta-analyses of mentoring programmes find an average effect size of 0.2 for young people’s behavioural and emotional outcomes [21, 22]. Typically, evaluations focus on mentoring interventions for adolescents and examine distal outcomes, or long-term consequences, such as reoffending and school grades.

The best-known and most frequently evaluated mentoring programme is Big Brothers Big Sisters of America (BBBSA), a community-based mentoring programme for disadvantaged 10–14 year-old children at risk of academic disengagement. BBBSA matches children to a volunteer adult, who is of the same gender and shares the same interests and goals as the mentored child, for at least 12 months of one-to-one mentoring. Randomised controlled trial (RCT) evaluations in the US demonstrate the effectiveness of BBBSA in improving behavioural and academic outcomes. For instance, mentored young people were 32% less likely to report hitting somebody during the previous 12 months, reported skipping 52% fewer days of school than non-mentored young people, and reported moderately better school grades (3% higher) than the control group [23, 24]. Additionally, mentored young people from minority ethnic backgrounds were 70% less likely to report initiating drug use [23, 24]. Furthermore, an RCT of Big Brothers Big Sisters in Ireland found that young people with a mentor felt more supported, showed more prosocial behaviour, and had a greater sense of hopefulness for the future than non-mentored young people [25].

While there are many variations of mentoring interventions, meta-analyses and research reviews have identified at least six features that are common to effective programmes. The first is matching the young person with the correct mentor [21, 22, 26–28]. A match based on shared interests (for example supporting the same football team) may make the young person more responsive to the adult’s guidance and advice, since those who perceive a high level of similarity tend to have higher-quality and longer-term relationships with their mentors. Second, mentoring programmes are more effective when there are structured activities planned, particularly ones that are driven by the needs and interests of the young person [21, 22, 26, 27]. Third, programmes targeted towards young people who are demonstrating behavioural difficulties tend to show greater impact than universal interventions [21, 22, 26]. Fourth, parent support and involvement in the programme is also beneficial [22, 26]. Fifth, the longer the mentoring relationship lasts, the better the outcome; relationships lasting for 12 months or longer have a more positive impact [26]. Sixth, the frequency of contact matters: one review found that programmes encouraging mentors and young people to meet at least once a week were more successful [17]. It is important for mentors to be clear about the frequency and duration of contact as this stops unrealistic expectations and allows a trusting, stable relationship to be built [22].

Although not researched as thoroughly, there are elements of mentoring programmes that reduce the chances of success. The main problem is a mismatch between the mentor and the young person [29]. Matching mentors and young people solely on the basis of race or ethnicity (something which often occurs) is not associated with improved outcomes [21]. Relationships that last for under 3 months can actually have a negative effect on young people’s confidence and self-worth [26]. Lack of mentor training and expertise has also been shown to decrease the effectiveness of mentoring [29].

### Chance UK’s early intervention mentoring programme

Chance UK’s mentoring programme is for children aged 5–11 years who are reported to be displaying challenging behaviour and emotional problems at school and at home. It is designed to intervene early in the development of such problems and aims to prevent future antisocial and criminal behaviour by reducing associated risk factors (such as early problem behaviour, lack of a positive role model, and limited opportunities) and by promoting children’s strengths (such as decision-making and coping skills, social skills, and competencies such as academic, sporting or creative abilities). Chance UK uses a solution-focused approach [30, 31] to improve children’s behaviour throughout 1 year of one-to-one mentoring by trained, supervised volunteers. The programme’s core design is in line with the features of effective mentoring programmes identified by meta-analyses and research reviews: Chance UK only serves children with an identified level of need; volunteer mentors are highly trained to deliver a tailored programme of structured activities; a thorough matching process – based on the mentor’s personality and characteristics – is designed to create successful matches; the sessions take place weekly for 1 year; and parents are offered support as part of the programme. As such, it is reasonable to expect that Chance UK’s programme will have an effect size that is greater than the average cited above.

Other aspects of Chance UK’s programme are different from mentoring programmes that have been evaluated previously by RCT or quasi-experimental design studies. In particular, it works with a younger age group than typical mentoring programmes, so a greater impact may be expected as younger children’s behaviour may be more malleable before negative behaviours become embedded [32, 33]. Chance UK’s programme also focuses on achieving more proximal outcomes, or short-term consequences, such as better behaviour via improvements in self-esteem and self-efficacy, rather than targeting distal outcomes such as delinquency and school grades that are the typical focus of mentoring programmes. Generally, interventions have stronger effects on proximal than distal outcomes [34]. Together, these factors suggest that the Chance UK programme will produce a higher effect size than is typically found in evaluations of mentoring programmes.

The Chance UK mentoring programme was previously evaluated in a pre-post study involving 100 children who had received mentoring [35]. This evaluation looked at changes in the level of children’s behavioural and emotional functioning from the beginning to the end of the programme. The parent-rated Strengths and Difficulties (SDQ) Total Difficulties score was available for 99 children entering the programme; on average this score was 19.25 out of 40 (within the ‘abnormal’ range (≥17) in the three-band classification of SDQ scores: www.sdqinfo.com). After a year of mentoring, the scores for 92 children who had data available decreased to an average of 14.82 out of 40 (within the ‘borderline’ range of difficulties (scores of 14–16), a statistically significant improvement (t_(91)_ = 7.15, *p* < 0.001). The average teacher-rated SDQ Total Difficulties scores decreased from 23.41 to 16.48 (t_(85)_ = 8.07, *p* < 0.001).

Building on this preliminary evidence, this paper describes the protocol for an RCT evaluating the Chance UK mentoring programme.

## Methods

### Objectives

The objectives of the trial are:

1. To estimate whether offering the Chance UK mentoring programme has an effect on children’s behaviour and socio-emotional well-being in comparison to similar children who were not offered the programme.
2. To estimate whether the Chance UK mentoring programme has an effect on children’s self-esteem and self-efficacy, both of which are hypothesised mediators in the programme’s theory of change.
3. To describe the extent to which the Chance UK mentoring programme is implemented with fidelity to the programme design.

It is hypothesised that, when compared with children who were not offered mentoring (the control arm), children who are offered the mentoring programme (the intervention arm) will, at follow-up, demonstrate fewer emotional and behavioural difficulties (as reported by parent/carers) and higher self-esteem and self-efficacy (self-reported by children who were aged 8 years or above at baseline).

### Design

**Fig. 1:**
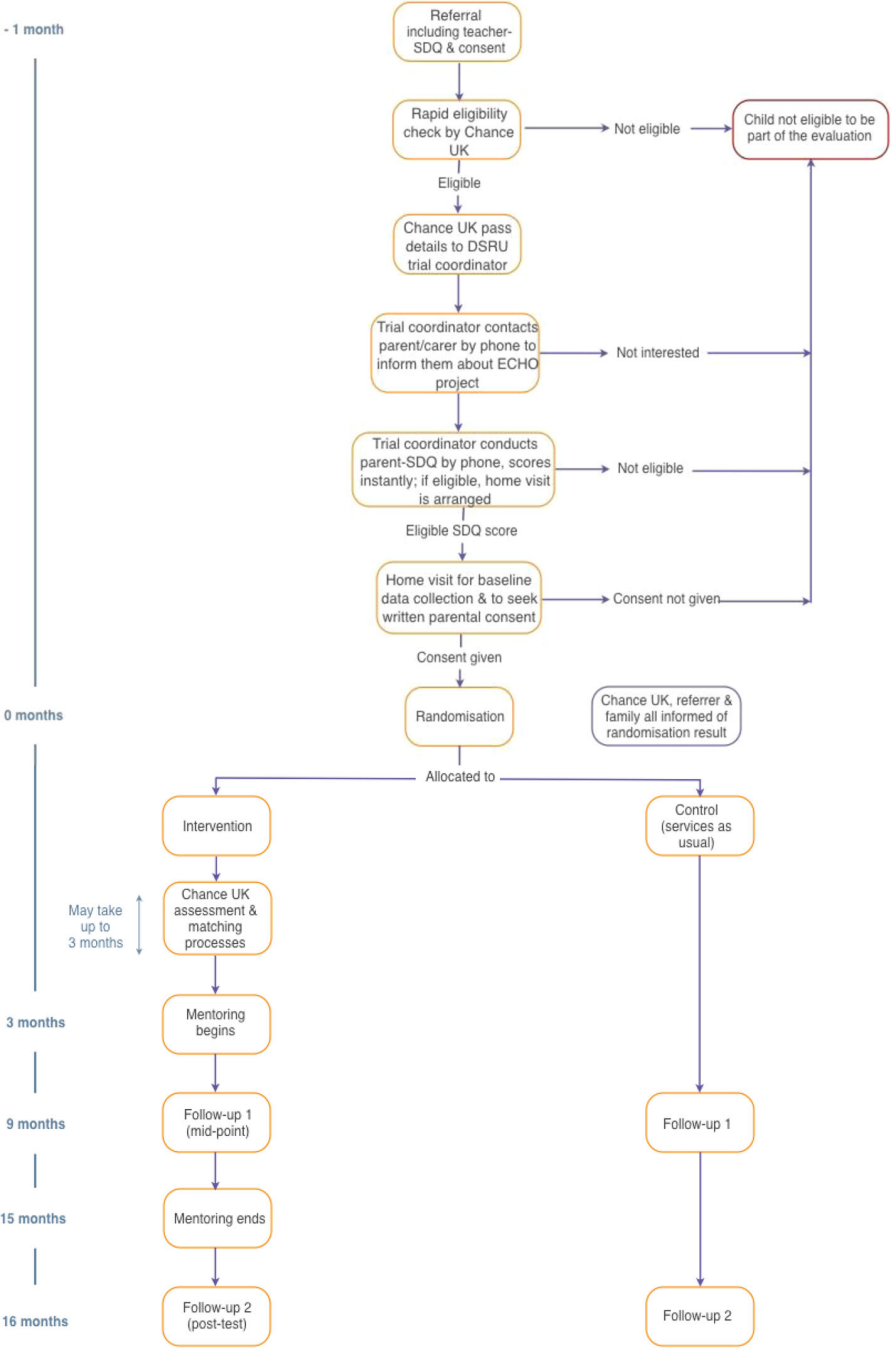
Trial timeline

A two-arm, randomised controlled, parallel group, superiority trial will be conducted to evaluate the effectiveness of Chance UK’s mentoring programme in improving behavioural and emotional outcomes in primary school children who have teacher- and parent/carer-reported behavioural difficulties. The intervention arm will be offered the mentoring programme; both trial arms will have access to services as usual. Assessments will take place pre-intervention (baseline), mid-way through the mentoring year (c.9 months after randomisation, midpoint) and post- mentoring programme (c.16 months after randomisation, endpoint). (See Fig. 1 for an overview of assessments.)

### Setting

The intervention will be delivered by Chance UK in a range of settings in the community in five boroughs of London, UK: Enfield, Hackney, Islington, Lambeth and Waltham Forest. Participants must live or attend school in one of these boroughs at the time of recruitment. The control group will come from the same population. Assessments for the RCT will take place in the home and school (online for teachers).

### Participants

Children are eligible to participate in the study if *all* of the following criteria are satisfied:

- The child is aged between 5 and 10 years old when referred to the project (meaning the child will be aged 5–11 while receiving mentoring).
- The child lives or attends school in any of the London boroughs of Enfield, Hackney, Islington, Lambeth or Waltham Forest.
- The child scores ≥16 on teacher-reported SDQ Total Difficulties (indicating that the child is in the ‘abnormal’ range).
- The child scores ≥14 on parent/carer-reported SDQ Total Difficulties (indicating that the child is in the ‘borderline’ (scores of 14–16) or ‘abnormal’ (scores of 17–40) range).
- Both parent/carer and child are willing to take part in the programme and the study (school staff understand that referral to the intervention constitutes referral to the study).
- There is no diagnosis of autism or any developmental delay that would prevent the child from engaging in the programme and the study, as identified through school records and parent report.
- There is no known risk of violence towards Chance UK staff or the research team by the child or parent/carer.
- The child does not have a sibling enrolled in the study.

### Recruitment and retention

Recruitment will take place between May 2014 and February 2016. Children will be referred to the trial by a member of school staff who knows the child well (e.g. a class teacher or Special Educational Needs Coordinator (SENCO)) and who has concerns about the child’s behaviour. Chance UK has well-established relationships with many primary schools in five London boroughs in which the service has been operating, and will be responsible for sourcing referrals from schools. New schools may be approached as part of Chance UK’s organisational strategy. Chance UK estimates that approximately 65 schools could potentially make a referral during the study period.

Analysis of a sample of Chance UK’s archive referral data suggests that around 5% of referrals will be ineligible based on criteria such as the child having autism spectrum disorder and/or developmental delay or the family being unsuitable for the programme. Of the remaining cases, about 10% will reach the ‘abnormal’ cut-off (≥16) on the teacher-rated Strengths and Difficulties Questionnaire [4]. The Chance UK case analysis showed that only 70% of teacher-rated eligible children also reach the eligibility threshold of the ‘borderline’ cut-off (≥14) on the parent/carer-rated SDQ. It is also assumed, based on a previous evaluation of a mentoring programme [36], that approximately 10% of the families who are referred and eligible will not be interested in taking part in the study and will not complete the baseline assessment. Chance UK must over-recruit to take these factors into account.

Communications about the research study will be distributed to schools directly by Chance UK and posted online alongside the referral form on Chance UK’s website. In particular, an information leaflet for school staff will be provided to explain the details of the research study and to make clear that during the recruitment phase of the study any referral to the service constitutes a referral to the research study.

Chance UK will screen each completed referral form, which contains the teacher-rated SDQ, to check eligibility for the research study. Provided that the child’s main parent/carer provides verbal consent to the referrer and the referrer gives written consent for this data to be shared with the research team, each suitable referral will be passed to the Trial Coordinator at Dartington Social Research Unit (DSRU) who will contact the main parent/carer by telephone to explain more about Chance UK’s programme and the research study, and to conduct further eligibility checks, including the baseline parent-rated SDQ. Where parents/carers are interested in their child participating and the child meets the initial eligibility criteria, an appointment will be made for an independent data collector to visit the family home to obtain written informed consent and collect additional baseline measures prior to randomisation.

Several strategies designed to minimise the level of attrition from the trial will be put in place. First, efforts will be made during the consent process and via information leaflets to make sure that participants are fully aware of what the research study involves and what will be expected, and to emphasise the value of taking part in the study. Second, the trial has been branded the ECHO project (Evidence for CHildren’s Outcomes) and will be communicated in a professional and attractive way that participants will be more likely to identify with and be interested in. Third, participants will be provided with change of address cards to notify the research team of new contact details. Fourth, efforts will be made to keep participants engaged in the study between data collection time points by sending a regular newsletter on the progress of the trial and a birthday card for the child. Fifth, families will be offered a small monetary incentive (shopping vouchers valued at £10) for each of the three home data collection appointments to compensate for their time spent in completing the questionnaires. Finally, Chance UK will work to keep school staff engaged and to support referrals to the project.

Should intervention group participants wish to withdraw from the mentoring programme, they will be encouraged to remain engaged in the research study by continuing to provide outcome data during assessment periods. Parents/carers will be informed of their right to withdraw their child from the research study at any time without giving any reason and with no adverse consequences; withdrawing from the study would not affect provision of the mentoring programme for the intervention group. Where parents/carers wish to fully withdraw their child from the study, *all* data collection for this case will cease (i.e. data will no longer be collected from teachers for the child). All previously collected data relating to this child will stand unless the parent/carer also asks for all them to be removed from the dataset (this can be done up to the point that data are analysed). Data collection with all participants will be completed voluntarily. Where parents/carers, school staff or children (aged 8–11) decline to complete a data collection point or request to withdraw themselves only, the assessments with the other reporters may still take place. For instance, a teacher may withdraw from the study but the assessments with the parent/carer and the child may continue, or the parent/carer may decline to complete a data collection point but data will still be collected from the teacher.

### Sample size

Recruitment of 246 eligible children to the project will allow detection of an effect size of 0.4 at *p* < 0.05 with 80% power (an effect size of 0.4 requires a minimum sample size of 99 participants per arm) and allows a study drop-out rate of up to 20%.

### Randomisation

A computer-generated randomisation sequence will be used to assign the participants to the intervention and control arms in a 1:1 ratio. Separate randomisation lists will be created for each site (Enfield, Hackney, Islington, Lambeth and Waltham Forest). In each location the first 25% of children will be allocated by simple randomisation and thereafter minimisation will be used to reduce imbalance between the programme and control groups in terms of age (< 9 versus ≥9 years) and gender (male versus female; the authors are not aware of any best practice recommendations on how to balance allocation for non-binary genders). Randomisation will take place after baseline data collection with families. The randomisation approach will be dynamic, meaning that each participant can be randomised as soon as they have completed the baseline assessments. The allocation sequence will be concealed using an online central randomisation service set up and maintained by statisticians at the University of Exeter (RT and TE, neither of whom are able to influence the data or the data analysis) that will conceal the sequence until assignment to group. The randomisation process will require the Trial Coordinator to log into a password-protected website and enter the relevant data of each newly recruited participant in order to receive the allocation.

### Blinding

Following randomisation, the Trial Coordinator will notify Chance UK, the child’s family and the referrer about the group allocation. The Principal Investigator, Trial Manager, data collectors and statisticians will be blind to participant allocation status. Allocation status will be recorded in a password-protected spreadsheet. The Trial Manager will be informed of allocation status if this is required to respond appropriately to a safeguarding concern (but will not be informed of the research ID for the child unless this is necessary).

Participants will be instructed not to reveal their allocation status to the data collector at the follow-up assessment points. It is considered unlikely that unblinding data collectors at any point in the study will bias the outcome data, as the outcome data are collected using self-completion questionnaires rather than through observation or interview (unless a participant asks the data collector to administer the questionnaires in interview style). After follow-up data collection, the data collector will be asked to report (i) whether they believe they know the allocation outcome and, if so, (ii) which arm they believe it to be and (iii) at which point during the visit they believe they were unblinded. If the data collector indicates that they believe they know the allocation outcome at midpoint, a different data collector will be asked to complete data collection with this family at endpoint (regardless of whether the suspicion is correct).

### Control arm

Children assigned to the control arm will receive services as usual, because the aim of the trial is to determine whether the mentoring programme provides added value. Chance UK state that the services on offer vary between boroughs and that services accessed by individual children will also vary. The offer is likely to include services and/or voluntary groups such as clubs, scouts, after school activities, CAMHS (Child and Adolescent Mental Health Services) and youth projects. Other services are unlikely to be highly similar to the Chance UK intervention, as reconnaissance suggests that typically few, if any, mentoring programmes are available in the relevant boroughs. Any services that children do receive, including other mentoring programmes, will be captured in a service use questionnaire (see below). In addition, referrers will be signposted to a standard universal children’s services directory available to each London borough that may be used to refer children to other services.

### Intervention arm

Children in the intervention arm will be offered the Chance UK mentoring programme. This comprises weekly one-to-one mentoring sessions over 1 year. Sessions last for 2 to 4 h and are tailored to each child. Mentors develop an individual programme of activities in line with their child’s interests and needs – this could include visits to the park, sports centre, library or exhibitions. All tasks are intended to be interactive and have a purpose: the aim of sessions is to help children progress to their identified ‘preferred future’ by working towards specified personal goals, to recognise and build strengths, and to consider and try out more effective responses to difficulties, all while broadening their horizons.

During mentoring sessions, the mentor uses techniques based on the solution-focused approach to help the child improve their behaviour without exploring the behaviour’s root cause. Instead, the focus is on building the child’s inner resources through developing personal and social skills crucial for dealing with frustration and conflict that once would have triggered an antisocial or inappropriate response.

Solution-focused techniques comprise the following four core components of the mentoring programme:

1. *Problem-free talk.* Language is purposely framed positively in order to create an environment where mentor and child are able to enjoy talking about shared interests, achievements and strengths without focusing on difficulties (which may often be the focus of children’s usual conversations with professionals). This allows the child to enjoy *problem-free time* with their mentor.
2. *Identifying and encouraging children’s strengths.* This component includes several techniques. One widely used technique is *finding exceptions*, where children are encouraged to challenge the negative statements they make about themselves based on their previous experiences. For example, if a child says “I am no good at anything”, the mentor will support them to identify a time when they did well. A second technique involves asking children *coping questions* to discuss what they did to cope with a difficult situation they experienced recently, and what stopped the situation from getting worse.
3. *Giving positive feedback.* Positive feedback is specific, identifying what the child has done well in a particular situation during the session. Specific feedback, rather than a general comment on their overall behaviour, builds self-esteem through highlighting strengths and helps the child to understand what they have done well, making them more likely to replicate this behaviour.
4. *Imagining a preferred future.* Tools under this heading help children to identify where they are in relation to a particular issue (such as controlling anger) and where they want to be. An exercise known as *scaling* involves asking the child to rate their position in relation to the issue on a scale of 1 to 10 (with 10 being the best the situation could be), eliciting information about what they have already done to get to this point, and then helping them to visualise and explain what a higher rating would look like and how it can be achieved.

The solution-focused approach is used alongside other strategies such as using star charts to highlight strengths and reward good behaviour.

The first 3 months of the programme form an ‘engagement period’, which focuses on building a trusting relationship between child and mentor and identifying the child’s difficulties and strengths. After 3 months, the mentor, child, main parent/carer and a member of Chance UK staff meet to set at least one behavioural goal, one educational or social skills goal and one fun goal. There are also often implicit goals that the mentor and Project Manager are more aware of than the child, such as helping the child to deal with anger. The rest of the mentoring year is focused on achieving these goals and building the child’s strengths. Each child may also choose to attend one or more group mentoring sessions with other children and mentors.

At the end of the mentoring year, all contact between the mentor and family must cease. After 9 months, therefore, the mentor and the child start preparing for a positive end to the mentoring relationship (the ‘endings process’). The end of the mentoring year is marked by a graduation ceremony that is attended by family and friends to celebrate successes and the goals achieved through the year. Chance UK conduct debrief sessions with the child, parent/carer, teachers and mentors to assess the effect of the mentoring on the child’s behaviour.

The theory of change for Chance UK’s intervention sets out how the core components of the intervention (described above) are designed to impact on children’s behaviour. The core components are designed to lead to improvements in children’s self-esteem, self-efficacy, social and relationship skills, positive coping skills, decision-making skills, aspirations and ability to regulate conduct and emotions. For example, giving positive feedback improves a child’s self-esteem, imagining a preferred future increases aspirations, and rewarding good behaviour encourages social and relationship skills. All of these factors can impact on a child’s behaviour [37], which is the primary focus of the intervention.

An intensive selection and training process involves recruiting mentors with the right qualities and skills, such as being a dedicated, focused and positive role model who is fun but also able to help the child stick to boundaries. The matching exercise pairs them with a child (as described earlier in this article).

In an optional part of the intervention, Chance UK can also work with the child’s parent(s) /carer(s), offering support, guidance and signposting, all aimed at maintaining positive changes in the child’s behaviour and stability for the family once the mentoring ends. The Chance UK Parent Programme is offered as an optional part of the mentoring intervention to all parents/carers of mentored children in boroughs where the programme is run and where there is funding for the parenting element. It is taken up by those who are interested. A Parent Programme Manager (PPM) contacts the family to explain the support that can be requested at any time during the mentoring year. The programme is flexible, supportive and non-judgmental. It involves applying the solution-focused approach in order to build a parent/carer’s self-confidence and ability to deal with any challenges they may face. A PPM (a member of Chance UK staff) is assigned to each family and the support they can provide is tailored to the needs of the family. It can range from low to high intensity, consisting of practical assistance with family management, for example budgeting or financial support to purchase necessary household items such as mattresses, or assisting with personal development such as preparing a CV, through to multi-agency and partnership working involving information sharing, representation, signposting and introduction to relevant universal and targeted services. Support can be offered through one-to-one sessions, family group sessions and/or group workshops that take place several times a year. The parent/carer service can take place throughout the mentoring programme but ends when the mentoring ends.

### Participant timeline

A schematic diagram of the participant timeline can be found in Fig. 1. A child is referred to Chance UK’s service by a member of school staff (e.g. a teacher or SENCO) who has completed the teacher-rated SDQ. Once assessed for eligibility, the remaining baseline assessments with the main parent/carer, and the child themselves if aged 8–11, will take place during two appointments: first by telephone to determine eligibility and interest in the programme and involvement in the research study; and second at a home visit to collect additional baseline data.

A case will be randomised once the participant has completed all baseline data collection. Follow-up data will be collected from all participants at two points: first, 9 months after the case was randomised (equivalent to mid-way through the mentoring year, given that the matching process can take up to 3 months), and second, 16 months after the case was randomised (equivalent to 1 month after the end of the mentoring year).

### Outcome measures

The parent SDQ Total Difficulties score is the primary outcome; all other outcomes described below are secondary.

#### Strengths and Difficulties Questionnaire (SDQ) [38]

The SDQ is a widely-used 25-item questionnaire with excellent psychometric properties for identifying children with behavioural and emotional difficulties in clinical and community populations [39, 40]. Versions of the questionnaire have been developed for self-report, completion by a parent/carer and completion by teachers. This study will include the Parent-report SDQ (PSDQ) and the Teacher-report SDQ (TSDQ) for children aged 4–17 years. The PSDQ and TSDQ each contain five subscales of five items, assessing conduct problems, emotional problems, hyperactivity, peer problems and prosocial behaviour respectively. Each item has three response options: 0 = not true; 1 = somewhat true; and 2 = certainly true. The hyperactivity, emotional, conduct, and peer problems subscales are summed to provide a Total Difficulties score with a possible range of 0 to 40, where higher scores indicate greater difficulties. Using the original three-band classification system for the SDQ, this score can be categorised into ‘Normal’ (0–13 PSDQ, 0–11 TSDQ), ‘Borderline’ (14–16 PSDQ, 12–15 TSDQ) and ‘Abnormal’ (17–40 PSDQ, 16–40 TSDQ).

The SDQ also includes a brief Impact Supplement, designed to capture the impact of behavioural and/or socio-emotional difficulties on the child, their everyday life and the people around them. Both the PSDQ and TSDQ Impact Supplement ask the respondent whether they consider the child to have difficulties in at least one domain assessed by the SDQ, with four response options (No; Yes – minor difficulties; Yes – definite difficulties; and Yes – severe difficulties). Where the respondent indicates ‘No’, the Impact Score is calculated as 0. If the respondent indicates that they consider the child to have difficulties in at least one of these domains, they are asked how long the difficulties have been present (Less than a month; 1 to 5 months; 6 to 12 months; Over a year) and whether the difficulties upset or distress the child (Not at all = 0; Only a little = 0; Quite a lot = 1; A great deal = 2).

The PSDQ Impact Supplement then asks whether the difficulties interfere in the child’s everyday life in four areas (Home life; Friendships; Classroom learning; Leisure activities) with four response options for each area (Not at all = 0; Only a little = 0; Quite a lot = 1; A great deal = 2) and using the same four response options whether the difficulties put a burden on the respondent or the family. Similarly, the TSDQ Impact Supplement asks whether the difficulties interfere in the child’s everyday life in two areas (Peer relationships; Classroom learning), with four response options for each area (Not at all = 0; Only a little = 0; Quite a lot = 1; A great deal = 2) and using the same four response options whether the difficulties put a burden on the respondent or the class.

The PSDQ and TSDQ Impact Scores are calculated by summing responses to whether the difficulties upset or distress the child, and whether they interfere in everyday life in each of the assessed areas. As such, the PSDQ Impact Score ranges from 0 to 10, and the TSDQ Impact Score ranges from 0 to 6, with a higher score indicating a greater impact.

The primary outcome is the PSDQ Total Difficulties score assessed at the endpoint. It was decided to use this rather than the teacher-rated version because the respondent is more likely to be consistent (i.e. the same person) across data collection points over 16 months.

The PSDQ Total Difficulties score at midpoint and the TSDQ Total Difficulties score at midpoint and endpoint will be secondary outcomes. The PSDQ Impact Score and TSDQ Impact Score (assessed at midpoint and endpoint only) will be used as secondary outcomes, assessed at endpoint, as will the five PSDQ and TSDQ subscales.

#### Eyberg Child Behaviour Inventory (ECBI) [41]

The ECBI is a 36-item parent/carer-rated measure of behaviour problems exhibited by children aged 2 to 16 years, with two scales: an Intensity Scale with a range of possible scores from 36 to 252, and a Problem Scale with a range of possible scores from 0 to 36. The Intensity Scale asks parent/carers to indicate the current frequency of 36 common behaviours on a 7-point response scale (1 = Never to 7 = Always) (Intensity score) and the Problem Scale asks whether each behaviour is considered to be problematic (Yes / No) (Problem score). The ECBI has good validity for internalising and externalising behaviour problems when compared with the Child Behaviour Checklist [42]. The Intensity score and Problem score will be used as secondary outcomes, assessed at midpoint and endpoint.

#### The Self-Perception Profile for Children (SPPC) [43]

The SPCC is a 36-item self-report measure comprising the following six-item scales: global self-worth; scholastic competence; athletic competence; social competence; physical appearance; and behavioural conduct. For each item, children are asked to read two contrasting statements (e.g. “Some kids do things they know they shouldn’t do BUT Other kids hardly ever do things they know they shouldn’t do.”) and identify which statement is most like them. Children are then instructed to indicate if the statement is “Really true for me” or “Sort of true for me” Response items are scored on a 4-point scale, where 1 or 2 represent “Really true for me” or “Sort of true for me” respectively in relation to lower self-perceived competency, and 3 or 4 represent “Sort of true for me” or “Really true for me” respectively in relation to higher self-perceived competency. The scale score is obtained by calculating the mean response score for the relevant items, with scores ranging from 1 to 4 for each scale. The measure has been shown to have good internal consistency (Cronbach’s alphas for each subscale are around 0.80) and to correlate (around 0.60) with comparable subscales on the Self-Description Questionnaire.

The SPCC has been used in previous evaluations of mentoring programmes, for example Big Brothers Big Sisters [24]. The global self-worth, scholastic competence, social competence and behavioural conduct scales will be used as secondary outcomes, all assessed at endpoint. Two scales (physical appearance and athletic competence) will not be used since the mentoring programme does not target self-esteem in these areas.

#### Children’s Hope Scale (CHS) [44]

The Children’s Hope Scale (CHS) is a six-item self-report measure with two three-item subscales, assessing whether children feel able to initiate and move towards goals (agency subscale) and whether children feel able to create a plan to work towards their goals (pathway subscale). The six items in the CHS are scored on a 6-point response scale (1 = None of the time to 6 = All of the time). The overall score is calculated by adding the responses to the six items; the subscales are not intended to be analysed separately since the construct of hope is theorised to consist of both elements. The measure has been shown to have good internal consistency (Cronbach alphas ranging from 0.72 to 0.86) and test-re-test reliability (correlations around 0.70), along with good validity, for example positive and significant correlations with subscales on the Harter Self-Perception Profile [44]. A modified version of the CHS was used in a previous evaluation of mentoring [25]. The CHS score will be used as a secondary outcome, assessed at endpoint.

### Other measures

#### Family Demographics Questionnaire (FDQ)

The study will use a short questionnaire to gather basic demographic information about the child and their family. It is adapted from one used in the trial of a parenting intervention [45] and includes variables such as date of birth, age, gender, ethnicity, SEN status, education, members of household, relationship quality, family health and financial situation. The data will be used to describe the sample, examine the extent to which demographic characteristics are balanced between trial arms and carry out attrition analyses (i.e. the extent to which participants who drop out from the intervention and control arms are different on variables such as gender, ethnicity, family type and deprivation). The FDQ will be administered at baseline.

#### Family Service Use Questionnaire (FSUQ)

The study will use a short questionnaire based on the Client Service Receipt Inventory (CSRI). The CSRI has been used in over 100 studies since it was first developed in the mid-1980s [46, 47]. The FSUQ will record the receipt of targeted school services and additional services, detailing the typical length and number of contacts. It will be used to assess what other services participants in the trial receive and in particular what participants in the control arm receive, as this will help to explore the trial results (for example, if there is no impact, whether it could be because of the services that control arm participants received). The FSUQ will be administered to the parent/carer at midpoint and endpoint.

#### Beck Depression Inventory II (BDI-II) Short Form [48]

The BDI-II Short Form is a self-report 13-item questionnaire which assesses cognitive-affective symptoms using a subscale from the BDI-II, a widely used measure for detecting depression. There is some evidence to suggest that maternal depression is associated with a tendency for mothers to over-report child behaviour problems [49, 50]. Thirteen items are presented in groups of 4 statements from which the parent/carer must select the one that best describes how they have been feeling over the past 2 weeks. Items cover areas such as sadness, loss of pleasure, self-dislike and crying. The scale is widely used and has good internal validity (alpha 0.81) [51]. The BDI-II Short Form will be administered at baseline, midpoint and endpoint; the score will be used as a covariate to detect whether parental/carer depression affects the way that parents/carers perceive and report on their child’s behavioural difficulties.

### Mentor demographics

Chance UK will record the gender, age, ethnicity and employment status of mentors.

### Intervention fidelity

Fidelity monitoring tools have been developed by Chance UK in association with the research team in order to monitor and promote the high-quality delivery of mentoring, including adherence to the core design of the programme. The fidelity monitoring process will be implemented and managed by Chance UK, who will share the data with DSRU for research purposes.

The tools include:

1. *Quality, Adherence, Dose (QAD) rating by Programme Managers*: After each mentoring session, mentors complete a self-report adherence checklist, which captures: the range of core components delivered; the number and length of sessions; the level of the child’s engagement; and further qualitative information about the mentoring session. Following each monthly supervision session, the Programme Manager completes a rating scale assessing the quality of the mentor’s delivery of the programme, taking into account the mentor’s recent self-reported adherence and discussion during supervision sessions. The Programme Manager’s ratings capture important aspects of the quality of the mentoring relationship, the mentor’s use of solution-focused techniques, work towards achieving the child’s goals and the extent to which the mentor engages with supervision and requirements of the programme. Each criterion is scored on a scale of 1–3 (where 1 means ‘Improvement needed’ and 3 means ‘Good’), meaning that the mentor can achieve a score between 13 and 39 for each QAD record, with up to 13 records completed over the mentoring year (the mean score will be used for fidelity analyses). The form also records the number and length of sessions. If an individual session is cancelled the mentor will record the reason for this on the Mentor Session Report form.
2. *The mentor’s perspective of the quality of support provided by Programme Managers*: A short quality assurance survey at the 4-month and 9-month time-points asks mentors for feedback on the support and guidance they receive as part of the supervision process and monitors whether this is of an appropriate standard. Mentors can respond ‘Always’, ‘Sometimes’ or ‘Never’ to questions such as ‘Do you feel your Programme Manager is sufficiently available to you for supervision, as well as extra support when needed?’. There are seven questions, and a mean score will be calculated for each mentor. The higher the score, the greater the level of perceived support and supervision. There are six Programme Managers at any one time.
3. *The child’s perspective of the quality of the mentor-child relationship*: This will be captured in the Mentor Youth Alliance Scale (MYAS) [52], administered to children by Programme Managers at 3 and 9 months into the mentoring year. The MYAS consists of 10 items in one scale focusing on positive aspects of the relationship. It has been shown to have good validity and reliability, notably a Cronbach’s alpha of 0.85 and positive correlation with the Adult Relationship Scale [52].
4. *Parent Work Record*: Not all parents/carers will sign up to the parent work and those who do so will receive varying levels of support. PPMs will update a form throughout the year to capture the total amount of contact hours provided and by what method (e.g. phone, face-to-face meeting) and the nature of support provided (selected from a range of themes, such as parent physical or mental health, financial issues, or parenting skills). Attendance at parent group sessions and family group sessions is also recorded.

### Data collection

Baseline data collection and randomisation will take place between July 2014 and March 2016. Midpoint data collection occurs 9-months post randomisation, and is therefore projected to take place between April 2015 and December 2016. Endpoint data collection occurs 16-months post randomisation and is projected to take place between November 2015 and July 2017.

### Statistical methods

Baseline and demographic characteristics will be summarised using means and standard deviations (or medians and interquartile ranges) for continuous variables and percentages for categorical variables. The comparison of the trial arms will use an intention-to-treat framework with participants analysed according to the trial arm they were randomised to, regardless of whether or not they received the intervention. The primary outcome is parent-reported child behaviour and emotional functioning derived from the SDQ Total Difficulties score (PSDQ) at endpoint. The secondary outcomes are: PSDQ Total Difficulties score (midpoint); PSDQ Total Difficulties score above the pre-specified clinically relevant threshold of 14 (endpoint); teacher-reported child behaviour and emotional functioning according to the TSDQ Total Difficulties score (midpoint and endpoint); the PSDQ and TSDQ Impact Supplement scores (endpoint); the PSDQ and TSDQ subscale scores (endpoint); the ECBI frequency and intensity scales (midpoint and endpoint); four SPPC subscales (endpoint); and the Children’s Hope Scale (endpoint).

The trial arms will be compared in crude (unadjusted) analyses presenting the mean difference for continuous outcomes and the odds ratio for binary outcomes. Linear regression (for continuous outcomes) and logistic regression (for binary outcomes) will be used to adjust these comparisons for the baseline score of the outcome in question, variables involved in randomisation (age group, gender and borough), ethnicity, SEN, SES and baseline BDI-II score. The adjusted and imputed (see below) analysis will be considered primary. Tests of interaction will be used to examine whether the effect of the intervention differs across categories based on age (< 9 versus ≥9 years), gender, ethnicity, level of total difficulties on the PSDQ (borderline < 16 vs. abnormal ≥17) at baseline, SES at baseline and lone parent (Yes/No) at baseline. The primary analyses will be based on analyses of 20 multiply imputed datasets to handle missing data.

Clustering may exist due to the sampling of children from the same school, although the majority of schools will likely provide only small numbers of children. A sensitivity analysis will be carried out by adding school as a random effect in analysis of the primary outcome (using a mixed effects model). If the primary outcome estimates are substantively affected by school, the random effect will then be entered in further analyses of outcome (primary and secondary).

Fidelity to the design of the intervention, including the individual mentoring, group mentoring and the parent work, will be summarised using descriptive statistics. It will be assessed in terms of the different dimensions measured (adherence, dose, quality and engagement). Regarding dose, full participation in individual mentoring requires that (i) children receive at least 35 sessions, and (ii) participation is over at least 11 months.

A secondary analysis will be undertaken to quantify the extent to which the intervention effect on the PSDQ Total Difficulties score at the endpoint (the primary outcome) is determined by participation in the intervention (number of months of mentoring received before the endpoint). A complier average causal effect analysis (CACE) [53, 54] will be undertaken on the complete case data.

### Participation

Participation in the research study by parents (and children) is voluntary. However, as a consequence of Chance UK only having capacity to serve those involved in the study, any family who is not willing at the outset to be involved in the research will not be eligible for Chance UK’s mentoring programme during the recruitment phase of the trial. This will be explained to potential participants. Once involved in the study, each data collection appointment is also completed voluntarily. School staff (referrers) will be made aware that referral to the service constitutes de facto referral to the research study, and the data they provide at referral will be used for the study purposes. Therefore, they should only make the referral if they voluntarily agree to this. School staff will also voluntarily complete each follow-up data collection appointment. Children in the intervention group will be able to continue receiving the mentoring programme once this has started, regardless of whether the family withdraws the child’s involvement in the research study.

### Informed consent

The referrer will provide written consent and ask the child’s parent/carer for verbal consent to make the referral and share information about the child with Chance UK and the research team at DSRU. The research team will affirm parental interest in participating in the trial and ask for verbal consent during contact with the parent/carer by telephone to complete the parent SDQ. An independent data collector (trained by the research team) will visit the family home to collect additional baseline data and obtain written informed consent from the main parent/carer before randomisation. (The main parent/carer will consent on behalf of their child to take part in the research study, as children in the study will all be below the age of 12 years.) If, during the course of a child’s involvement in the research, there is a change of who holds parental responsibility (for example if the child becomes looked after), informed consent for future data collection will be sought from the person or Local Authority subsequently holding parental responsibility. As the referrer may no longer be involved with the child at the follow-up points in the study, any teacher asked to complete the teacher-reported SDQ at follow-up will be asked to indicate their consent online or on paper.

Children who are eligible to complete questionnaires at baseline and follow-up assessments (i.e. if they were at least 8 years old at baseline) will be given a verbal explanation of the research by the data collector and a written assent form to sign.

### Withdrawal

Parents/carers will be informed of their right to withdraw their child from the research study at any time without giving any reason. All data collection relating to this case would then cease (i.e. with school staff, the parent/carer and the child). All previously collected data relating to this child will still stand unless a parent/carer also asks for all this to be removed from the dataset (parents/carers will be informed that this is possible up to the point that the data is analysed). Where parents/carers, school staff or children (where applicable) wish to withdraw only themselves from the study in terms of completing questionnaires, the assessments with the other reporters will still take place.

All participants will be assured that there will be no adverse consequences of withdrawing from the study. Children in the intervention group will be able to continue receiving the mentoring programme once this has started, regardless of whether the family withdraws the child’s involvement in the research study.

### Confidentiality

All participants (school staff, parents/carers and children) will be informed that the data they provide will be treated confidentially. They will be made aware that in published reports the results will be reported anonymously and at a group level, meaning that it will not be possible to identify any individual or attribute any information to them. Parents/carers will be informed that if they disclose anything concerning child safety then the research team will be obliged to report this.

### Data sharing

Implementation fidelity data and programme evaluation data will be collected by Programme Managers at Chance UK in line with protocols laid out in the programme manual. A data sharing agreement between Chance UK and the research team will provide the research team with access to this data, and Chance UK with access to the SDQ data for the intervention arm which, under usual circumstances (i.e. were the trial not taking place), would have been collected by Chance UK Programme Managers.

## Discussion

This RCT will be instrumental in building the UK evidence base for early intervention mentoring programmes. In particular, it will examine the impact of an intensive mentoring programme (weekly sessions over a 12-month duration) with primary school-aged children who are demonstrating significant behavioural and/or emotional difficulty. The project also offers the opportunity to demonstrate that the utilisation of RCTs to evaluate social interventions in real world, third-sector settings is both achievable and valid.

## Abbreviations

BBBSA: Big Brothers Big Sisters of America
BDI-II: Beck Depression Inventory II
CACE: Complier Average Causal Effect
CAMHS: Child and Adolescent Mental Health Services
CHS: Children’s Hope Scale
CSRI: Client Service Receipt Inventory
DSRU: Dartington Social Research Unit
ECBI: Eyberg Child Behaviour Inventory
ECHO: Evidence for Children’s Outcomes
FDQ: Family Demographics Questionnaire
FSUQ: Family Service Use Questionnaire
PPM: Parent Programme Manager
PSDQ: Parent version of the Strengths and Difficulties Questionnaire
QAD: Quality, Adherence, Dose
RCT: Randomised controlled trial
SDQ: Strengths and Difficulties Questionnaire
SEN: Special educational needs
SENCO: Special Educational Needs Coordinator
SES: Socio-economic status
SPPC: Self-Perception Profile for Children
TSDQ: Teacher version of the Strengths and Difficulties Questionnaire
